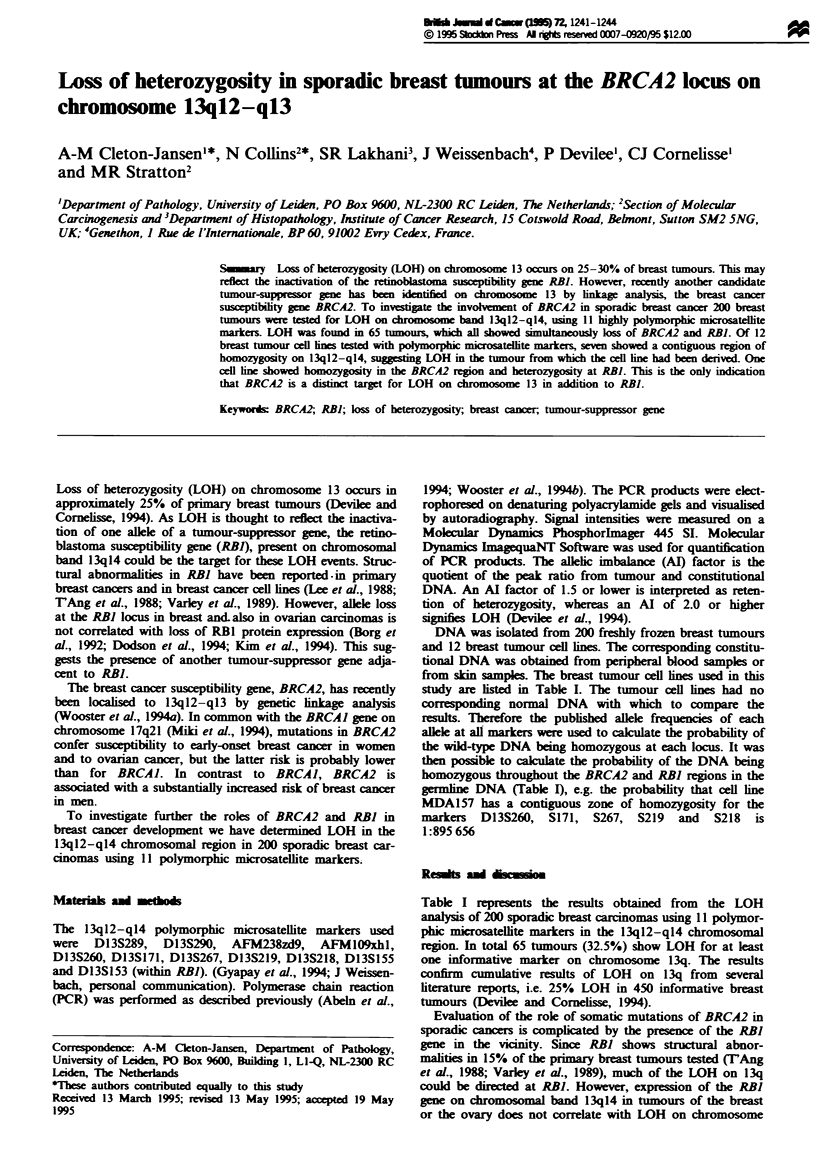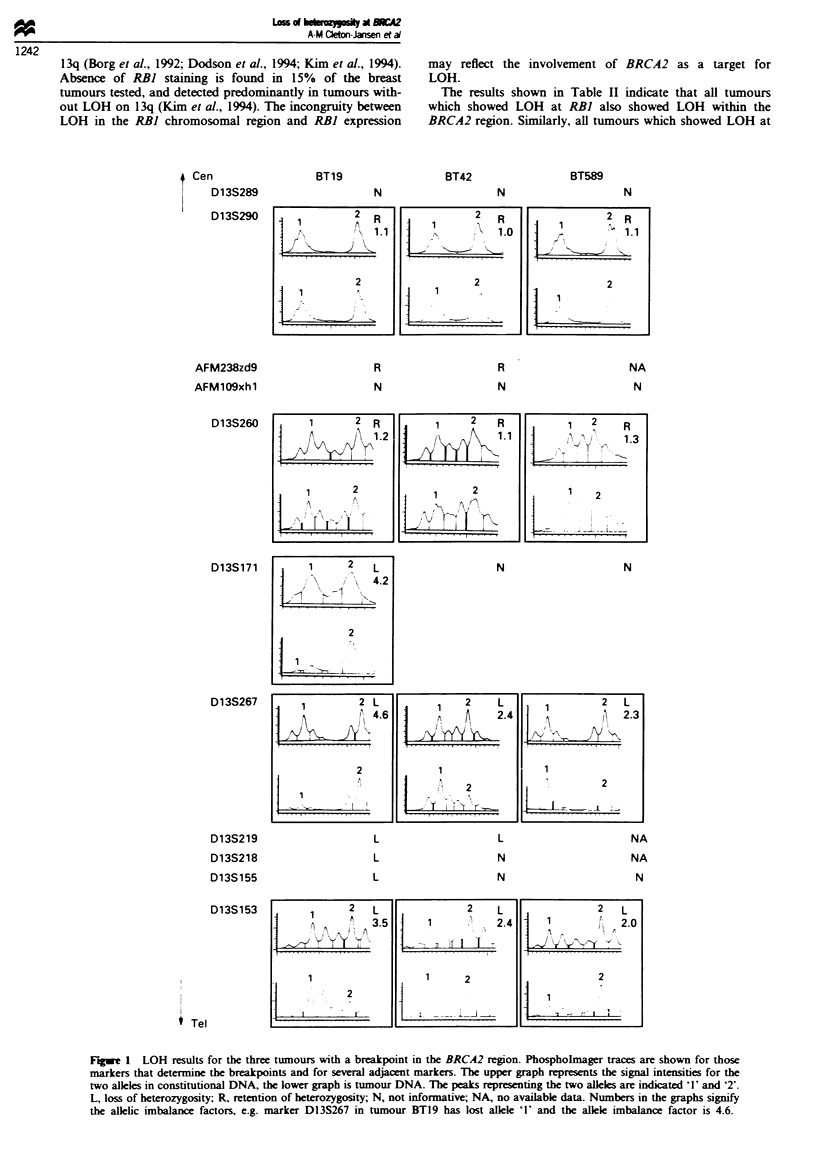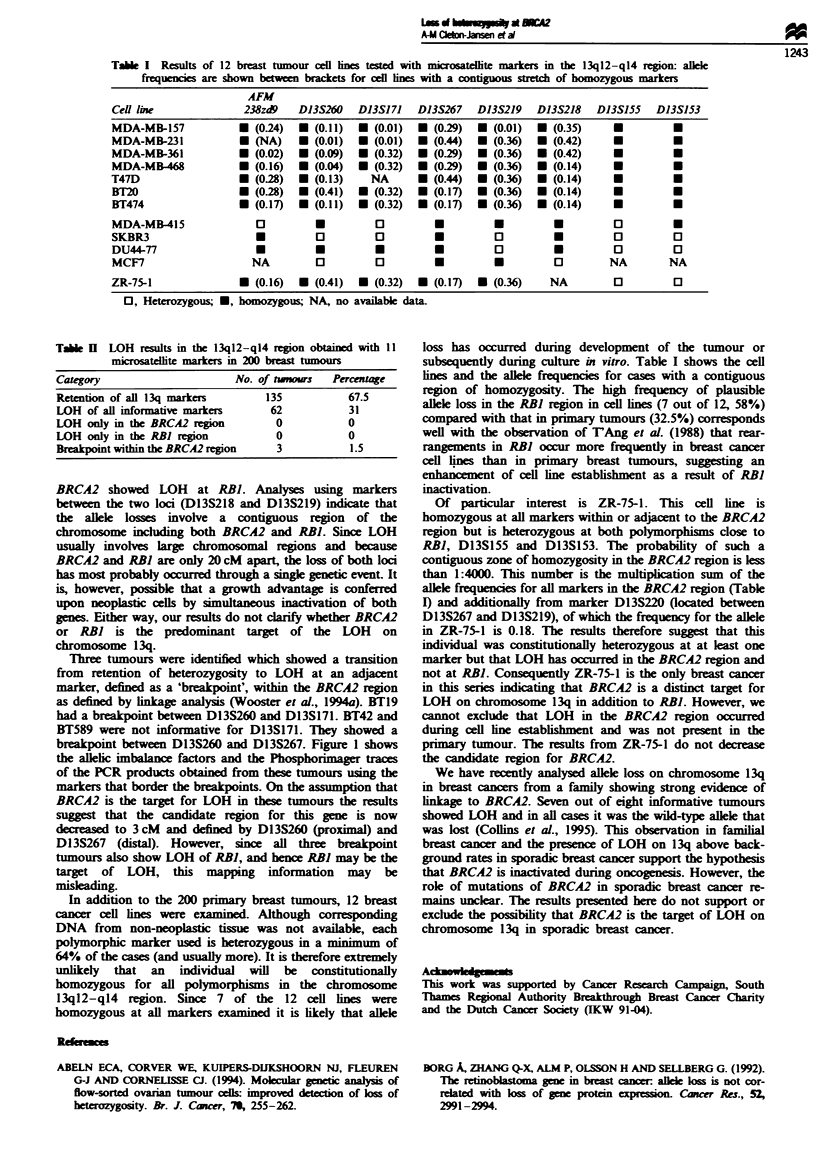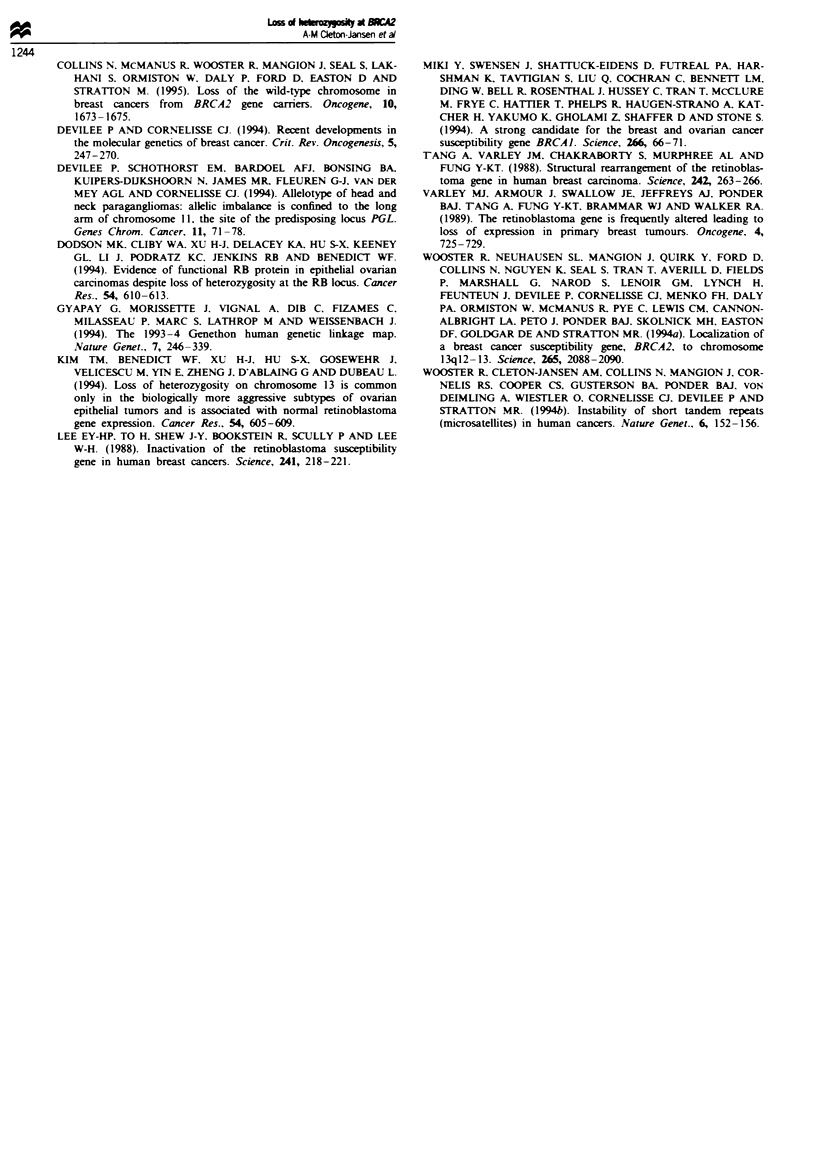# Loss of heterozygosity in sporadic breast tumours at the BRCA2 locus on chromosome 13q12-q13.

**DOI:** 10.1038/bjc.1995.493

**Published:** 1995-11

**Authors:** A. M. Cleton-Jansen, N. Collins, S. R. Lakhani, J. Weissenbach, P. Devilee, C. J. Cornelisse, M. R. Stratton

**Affiliations:** Department of Pathology, University of Leiden, The Netherlands.

## Abstract

Loss of heterozygosity (LOH) on chromosome 13 occurs on 25-30% of breast tumours. This may reflect the inactivation of the retinoblastoma susceptibility gene RB1. However, recently another candidate tumour-suppressor gene has been identified on chromosome 13 by linkage analysis, the breast cancer susceptibility gene BRCA2. To investigate the involvement of BRCA2 in sporadic breast cancer 200 breast tumours were tested for LOH on chromosome band 13q12-q14, using 11 highly polymorphic microsatellite markers. LOH was found in 65 tumours, which all showed simultaneously loss of BRCA2 and RB1. Of 12 breast tumour cell lines tested with polymorphic microsatellite markers, seven showed a contiguous region of homozygosity on 13q12-q14, suggesting LOH in the tumour from which the cell line had been derived. One cell line showed homozygosity in the BRCA2 region and heterozygosity at RB1. This is the only indication that BRCA2 is a distinct target for LOH on chromosome 13 in addition to RB1.


© 1995 Stockton Press All rights reserved 0007-0920/95 $12.00

### Loss of heterozygosity in sporadic breast tumours at the *BRCA2* locus on chromosome 13q12-q13

A-M Cleton-Jansen[1]*, N Collins[2]*, SR Lakhani[3], J Weissenbach[4], P Devilee[1], CJ Cornelisse[1] and MR Stratton[2]

*[1]Department of Pathology, University of Leiden, PO Box 9600, NL-2300 RC Leiden, The Netherlands; [2]Section of Molecular Carcinogenesis and [3]Department of Histopathology, Institute of Cancer Research, 15 Cotswold Road, Belmont, Sutton SM2 5NG, UK; [4]Genethon, 1 Rue de l'Internationale, BP 60, 91002 Evry Cedex, France.*

**Summary** Loss of heterozygosity (LOH) on chromosome 13 occurs on 25-30% of breast tumours. This may reflect the inactivation of the retinoblastoma susceptibility gene *RB1*. However, recently another candidate tumour-suppressor gene has been identified on chromosome 13 by linkage analysis, the breast cancer susceptibility gene *BRCA2*. To investigate the involvement of *BRCA2* in sporadic breast cancer 200 breast tumours were tested for LOH on chromosome band 13q12-q14, using 11 highly polymorphic microsatellite markers. LOH was found in 65 tumours, which all showed simultaneously loss of *BRCA2* and *RB1*. Of 12 breast tumour cell lines tested with polymorphic microsatellite markers, seven showed a contiguous region of homozygosity on 13q12-q14, suggesting LOH in the tumour from which the cell line had been derived. One cell line showed homozygosity in the *BRCA2* region and heterozygosity at *RB1*. This is the only indication that *BRCA2* is a distinct target for LOH on chromosome 13 in addition to *RB1*.

**Keywords:** *BRCA2*; *RB1*; loss of heterozygosity; breast cancer; tumour-suppressor gene

Loss of heterozygosity (LOH) on chromosome 13 occurs in approximately 25% of primary breast tumours (Devilee and Cornelisse, 1994). As LOH is thought to reflect the inactivation of one allele of a tumour-suppressor gene, the retinoblastoma susceptibility gene (*RB1*), present on chromosomal band 13q14 could be the target for these LOH events. Structural abnormalities in *RB1* have been reported in primary breast cancers and in breast cancer cell lines (Lee *et al.*, 1988; T'Ang *et al.*, 1988; Varley *et al.*, 1989). However, allele loss at the *RB1* locus in breast and also in ovarian carcinomas is not correlated with loss of RB1 protein expression (Borg *et al.*, 1992; Dodson *et al.*, 1994; Kim *et al.*, 1994). This suggests the presence of another tumour-suppressor gene adjacent to *RB1*.

The breast cancer susceptibility gene, *BRCA2*, has recently been localised to 13q12-q13 by genetic linkage analysis (Wooster *et al.*, 1994*a*). In common with the *BRCA1* gene on chromosome 17q21 (Miki *et al.*, 1994), mutations in *BRCA2* confer susceptibility to early-onset breast cancer in women and to ovarian cancer, but the latter risk is probably lower than for *BRCA1*. In contrast to *BRCA1*, *BRCA2* is associated with a substantially increased risk of breast cancer in men.

To investigate further the roles of *BRCA2* and *RB1* in breast cancer development we have determined LOH in the 13q12-q14 chromosomal region in 200 sporadic breast carcinomas using 11 polymorphic microsatellite markers.

### Materials and methods

The 13q12-q14 polymorphic microsatellite markers used were D13S289, D13S290, AFM238zd9, AFM109xh1, D13S260, D13S171, D13S267, D13S219, D13S218, D13S155 and D13S153 (within *RB1*). (Gyapay *et al.*, 1994; J Weissenbach, personal communication). Polymerase chain reaction (PCR) was performed as described previously (Abeln *et al.*, 1994; Wooster *et al.*, 1994*b*). The PCR products were electrophoresed on denaturing polyacrylamide gels and visualised by autoradiography. Signal intensities were measured on a Molecular Dynamics PhosphorImager 445 SI. Molecular Dynamics ImagequaNT Software was used for quantification of PCR products. The allelic imbalance (AI) factor is the quotient of the peak ratio from tumour and constitutional DNA. An AI factor of 1.5 or lower is interpreted as retention of heterozygosity, whereas an AI of 2.0 or higher signifies LOH (Devilee *et al.*, 1994).

DNA was isolated from 200 freshly frozen breast tumours and 12 breast tumour cell lines. The corresponding constitutional DNA was obtained from peripheral blood samples or from skin samples. The breast tumour cell lines used in this study are listed in Table I. The tumour cell lines had no corresponding normal DNA with which to compare the results. Therefore the published allele frequencies of each allele at all markers were used to calculate the probability of the wild-type DNA being homozygous at each locus. It was then possible to calculate the probability of the DNA being homozygous throughout the *BRCA2* and *RB1* regions in the germline DNA (Table I), e.g. the probability that cell line MDA157 has a contiguous zone of homozygosity for the markers D13S260, S171, S267, S219 and S218 is 1:895 656

### Results and discussion

Table I represents the results obtained from the LOH analysis of 200 sporadic breast carcinomas using 11 polymorphic microsatellite markers in the 13q12-q14 chromosomal region. In total 65 tumours (32.5%) show LOH for at least one informative marker on chromosome 13q. The results confirm cumulative results of LOH on 13q from several literature reports, i.e. 25% LOH in 450 informative breast tumours (Devilee and Cornelisse, 1994).

Evaluation of the role of somatic mutations of *BRCA2* in sporadic cancers is complicated by the presence of the *RB1* gene in the vicinity. Since *RB1* shows structural abnormalities in 15% of the primary breast tumours tested (T'Ang *et al.*, 1988; Varley *et al.*, 1989), much of the LOH on 13q could be directed at *RB1*. However, expression of the *RB1* gene on chromosomal band 13q14 in tumours of the breast or the ovary does not correlate with LOH on chromosome

Correspondence: A-M Cleton-Jansen, Department of Pathology, University of Leiden, PO Box 9600, Building 1, L1-Q, NL-2300 RC Leiden, The Netherlands

*These authors contributed equally to this study

Received 13 March 1995; revised 13 May 1995; accepted 19 May 1995

13q (Borg *et al.*, 1992; Dodson *et al.*, 1994; Kim *et al.*, 1994). Absence of *RB1* staining is found in 15% of the breast tumours tested, and detected predominantly in tumours without LOH on 13q (Kim *et al.*, 1994). The incongruity between LOH in the *RB1* chromosomal region and *RB1* expression may reflect the involvement of *BRCA2* as a target for LOH.

The results shown in Table II indicate that all tumours which showed LOH at *RB1* also showed LOH within the *BRCA2* region. Similarly, all tumours which showed LOH at

**Figure 1** LOH results for the three tumours with a breakpoint in the *BRCA2* region. PhosphoImager traces are shown for those markers that determine the breakpoints and for several adjacent markers. The upper graph represents the signal intensities for the two alleles in constitutional DNA, the lower graph is tumour DNA. The peaks representing the two alleles are indicated '1' and '2'. L, loss of heterozygosity; R, retention of heterozygosity; N, not informative; NA, no available data. Numbers in the graphs signify the allelic imbalance factors, e.g. marker D13S267 in tumour BT19 has lost allele '1' and the allele imbalance factor is 4.6.

**Table I** Results of 12 breast tumour cell lines tested with microsatellite markers in the 13q12–q14 region: allele frequencies are shown between brackets for cell lines with a contiguous stretch of homozygous markers

| *Cell line* | *AFM 238zd9* | *D13S260* | *D13S171* | *D13S267* | *D13S219* | *D13S218* | *D13S155* | *D13S153* |
|---|---|---|---|---|---|---|---|---|
| MDA-MB-157 | ■ (0.24) | ■ (0.11) | ■ (0.01) | ■ (0.29) | ■ (0.01) | ■ (0.35) | ■ | ■ |
| MDA-MB-231 | ■ (NA) | ■ (0.01) | ■ (0.01) | ■ (0.44) | ■ (0.36) | ■ (0.42) | ■ | ■ |
| MDA-MB-361 | ■ (0.02) | ■ (0.09) | ■ (0.32) | ■ (0.29) | ■ (0.36) | ■ (0.42) | ■ | ■ |
| MDA-MB-468 | ■ (0.16) | ■ (0.04) | ■ (0.32) | ■ (0.29) | ■ (0.36) | ■ (0.14) | ■ | ■ |
| T47D | ■ (0.28) | ■ (0.13) | NA | ■ (0.44) | ■ (0.36) | ■ (0.14) | ■ | ■ |
| BT20 | ■ (0.28) | ■ (0.41) | ■ (0.32) | ■ (0.17) | ■ (0.36) | ■ (0.14) | ■ | ■ |
| BT474 | ■ (0.17) | ■ (0.11) | ■ (0.32) | ■ (0.17) | ■ (0.36) | ■ (0.14) | ■ | ■ |
| MDA-MB-415 | □ | ■ | □ | ■ | ■ | ■ | □ | ■ |
| SKBR3 | ■ | □ | □ | ■ | □ | ■ | □ | □ |
| DU44-77 | ■ | ■ | ■ | ■ | □ | ■ | □ | □ |
| MCF7 | NA | □ | □ | ■ | ■ | □ | NA | NA |
| ZR-75-1 | ■ (0.16) | ■ (0.41) | ■ (0.32) | ■ (0.17) | ■ (0.36) | NA | □ | □ |

□, Heterozygous; ■, homozygous; NA, no available data.

**Table II** LOH results in the 13q12–q14 region obtained with 11 microsatellite markers in 200 breast tumours

| *Category* | *No. of tumours* | *Percentage* |
|---|---|---|
| Retention of all 13q markers | 135 | 67.5 |
| LOH of all informative markers | 62 | 31 |
| LOH only in the *BRCA2* region | 0 | 0 |
| LOH only in the *RB1* region | 0 | 0 |
| Breakpoint within the *BRCA2* region | 3 | 1.5 |

*BRCA2* showed LOH at *RB1*. Analyses using markers between the two loci (D13S218 and D13S219) indicate that the allele losses involve a contiguous region of the chromosome including both *BRCA2* and *RB1*. Since LOH usually involves large chromosomal regions and because *BRCA2* and *RB1* are only 20 cM apart, the loss of both loci has most probably occurred through a single genetic event. It is, however, possible that a growth advantage is conferred upon neoplastic cells by simultaneous inactivation of both genes. Either way, our results do not clarify whether *BRCA2* or *RB1* is the predominant target of the LOH on chromosome 13q.

Three tumours were identified which showed a transition from retention of heterozygosity to LOH at an adjacent marker, defined as a 'breakpoint', within the *BRCA2* region as defined by linkage analysis (Wooster *et al.*, 1994*a*). BT19 had a breakpoint between D13S260 and D13S171. BT42 and BT589 were not informative for D13S171. They showed a breakpoint between D13S260 and D13S267. Figure 1 shows the allelic imbalance factors and the Phosphorimager traces of the PCR products obtained from these tumours using the markers that border the breakpoints. On the assumption that *BRCA2* is the target for LOH in these tumours the results suggest that the candidate region for this gene is now decreased to 3 cM and defined by D13S260 (proximal) and D13S267 (distal). However, since all three breakpoint tumours also show LOH of *RB1*, and hence *RB1* may be the target of LOH, this mapping information may be misleading.

In addition to the 200 primary breast tumours, 12 breast cancer cell lines were examined. Although corresponding DNA from non-neoplastic tissue was not available, each polymorphic marker used is heterozygous in a minimum of 64% of the cases (and usually more). It is therefore extremely unlikely that an individual will be constitutionally homozygous for all polymorphisms in the chromosome 13q12–q14 region. Since 7 of the 12 cell lines were homozygous at all markers examined it is likely that allele loss has occurred during development of the tumour or subsequently during culture *in vitro*. Table I shows the cell lines and the allele frequencies for cases with a contiguous region of homozygosity. The high frequency of plausible allele loss in the *RB1* region in cell lines (7 out of 12, 58%) compared with that in primary tumours (32.5%) corresponds well with the observation of T'Ang *et al.* (1988) that rearrangements in *RB1* occur more frequently in breast cancer cell lines than in primary breast tumours, suggesting an enhancement of cell line establishment as a result of *RB1* inactivation.

Of particular interest is ZR-75-1. This cell line is homozygous at all markers within or adjacent to the *BRCA2* region but is heterozygous at both polymorphisms close to *RB1*, D13S155 and D13S153. The probability of such a contiguous zone of homozygosity in the *BRCA2* region is less than 1:4000. This number is the multiplication sum of the allele frequencies for all markers in the *BRCA2* region (Table I) and additionally from marker D13S220 (located between D13S267 and D13S219), of which the frequency for the allele in ZR-75-1 is 0.18. The results therefore suggest that this individual was constitutionally heterozygous at at least one marker but that LOH has occurred in the *BRCA2* region and not at *RB1*. Consequently ZR-75-1 is the only breast cancer in this series indicating that *BRCA2* is a distinct target for LOH on chromosome 13q in addition to *RB1*. However, we cannot exclude that LOH in the *BRCA2* region occurred during cell line establishment and was not present in the primary tumour. The results from ZR-75-1 do not decrease the candidate region for *BRCA2*.

We have recently analysed allele loss on chromosome 13q in breast cancers from a family showing strong evidence of linkage to *BRCA2*. Seven out of eight informative tumours showed LOH and in all cases it was the wild-type allele that was lost (Collins *et al.*, 1995). This observation in familial breast cancer and the presence of LOH on 13q above background rates in sporadic breast cancer support the hypothesis that *BRCA2* is inactivated during oncogenesis. However, the role of mutations of *BRCA2* in sporadic breast cancer remains unclear. The results presented here do not support or exclude the possibility that *BRCA2* is the target of LOH on chromosome 13q in sporadic breast cancer.

**Acknowledgements**

This work was supported by Cancer Research Campaign, South Thames Regional Authority Breakthrough Breast Cancer Charity and the Dutch Cancer Society (IKW 91-04).

**References**

ABELN ECA, CORVER WE, KUIPERS-DIJKSHOORN NJ, FLEUREN G-J AND CORNELISSE CJ. (1994). Molecular genetic analysis of flow-sorted ovarian tumour cells: improved detection of loss of heterozygosity. *Br. J. Cancer*, **70**, 255–262.

BORG Å, ZHANG Q-X, ALM P, OLSSON H AND SELLBERG G. (1992). The retinoblastoma gene in breast cancer: allele loss is not correlated with loss of gene protein expression. *Cancer Res.*, **52**, 2991–2994.

COLLINS N, MCMANUS R, WOOSTER R, MANGION J, SEAL S, LAKHANI S, ORMISTON W, DALY P, FORD D, EASTON D AND STRATTON M. (1995). Loss of the wild-type chromosome in breast cancers from *BRCA2* gene carriers. *Oncogene*, **10**, 1673–1675.

DEVILEE P AND CORNELISSE CJ. (1994). Recent developments in the molecular genetics of breast cancer. *Crit. Rev. Oncogenesis*, **5**, 247–270.

DEVILEE P, SCHOTHORST EM, BARDOEL AFJ, BONSING BA, KUIPERS-DIJKSHOORN N, JAMES MR, FLEUREN G-J, VAN DER MEY AGL AND CORNELISSE CJ. (1994). Allelotype of head and neck paragangliomas: allelic imbalance is confined to the long arm of chromosome 11, the site of the predisposing locus *PGL*. *Genes Chrom. Cancer*, **11**, 71–78.

DODSON MK, CLIBY WA, XU H-J, DELACEY KA, HU S-X, KEENEY GL, LI J, PODRATZ KC, JENKINS RB AND BENEDICT WF. (1994). Evidence of functional RB protein in epithelial ovarian carcinomas despite loss of heterozygosity at the RB locus. *Cancer Res.*, **54**, 610–613.

GYAPAY G, MORISSETTE J, VIGNAL A, DIB C, FIZAMES C, MILASSEAU P, MARC S, LATHROP M AND WEISSENBACH J. (1994). The 1993–4 Genethon human genetic linkage map. *Nature Genet.*, **7**, 246–339.

KIM TM, BENEDICT WF, XU H-J, HU S-X, GOSEWEHR J, VELICESCU M, YIN E, ZHENG J, D'ABLAING G AND DUBEAU L. (1994). Loss of heterozygosity on chromosome 13 is common only in the biologically more aggressive subtypes of ovarian epithelial tumors and is associated with normal retinoblastoma gene expression. *Cancer Res.*, **54**, 605–609.

LEE EY-HP, TO H, SHEW J-Y, BOOKSTEIN R, SCULLY P AND LEE W-H. (1988). Inactivation of the retinoblastoma susceptibility gene in human breast cancers. *Science*, **241**, 218–221.

MIKI Y, SWENSEN J, SHATTUCK-EIDENS D, FUTREAL PA, HARSHMAN K, TAVTIGIAN S, LIU Q, COCHRAN C, BENNETT LM, DING W, BELL R, ROSENTHAL J, HUSSEY C, TRAN T, MCCLURE M, FRYE C, HATTIER T, PHELPS R, HAUGEN-STRANO A, KATCHER H, YAKUMO K, GHOLAMI Z, SHAFFER D AND STONE S. (1994). A strong candidate for the breast and ovarian cancer susceptibility gene *BRCA1*. *Science*, **266**, 66–71.

T'ANG A, VARLEY JM, CHAKRABORTY S, MURPHREE AL AND FUNG Y-KT. (1988). Structural rearrangement of the retinoblastoma gene in human breast carcinoma. *Science*, **242**, 263–266.

VARLEY MJ, ARMOUR J, SWALLOW JE, JEFFREYS AJ, PONDER BAJ, T'ANG A, FUNG Y-KT, BRAMMAR WJ AND WALKER RA. (1989). The retinoblastoma gene is frequently altered leading to loss of expression in primary breast tumours. *Oncogene*, **4**, 725–729.

WOOSTER R, NEUHAUSEN SL, MANGION J, QUIRK Y, FORD D, COLLINS N, NGUYEN K, SEAL S, TRAN T, AVERILL D, FIELDS P, MARSHALL G, NAROD S, LENOIR GM, LYNCH H, FEUNTEUN J, DEVILEE P, CORNELISSE CJ, MENKO FH, DALY PA, ORMISTON W, MCMANUS R, PYE C, LEWIS CM, CANNON-ALBRIGHT LA, PETO J, PONDER BAJ, SKOLNICK MH, EASTON DF, GOLDGAR DE AND STRATTON MR. (1994*a*). Localization of a breast cancer susceptibility gene, *BRCA2*, to chromosome 13q12–13. *Science*, **265**, 2088–2090.

WOOSTER R, CLETON-JANSEN AM, COLLINS N, MANGION J, CORNELIS RS, COOPER CS, GUSTERSON BA, PONDER BAJ, VON DEIMLING A, WIESTLER O, CORNELISSE CJ, DEVILEE P AND STRATTON MR. (1994*b*). Instability of short tandem repeats (microsatellites) in human cancers. *Nature Genet.*, **6**, 152–156.